# Response to Beale

**DOI:** 10.1192/bjb.2022.24

**Published:** 2022-06

**Authors:** A. Z. FRCPsych, B. Z. FRCPsych

**Affiliations:** Independent Scholar, UK; Independent Scholar, UK Email: c/o BJPBulletin@rcpsych.ac.uk

We write to add our personal experience of 30 years of consultant psychiatric practice and of having a son with a long history of serious psychiatric problems to endorse Chloe Beale's powerful indictment of current mental health service failures. Having found our son in his room 2 weeks ago attempting to hang himself, we called 999. The ambulance arrived within a few minutes; he sat for 9 hours in the ambulance outside A&E before being seen by a series of three mental health nurses with questionnaires and was then summarily sent to walk home, still actively suicidal, past a famous ‘suicide spot’. We were not informed that he had been discharged. We had rung to ask what was happening and were summarily dismissed by one of the nurses with the cryptic comment that he had been ‘signposted to the Road’ (apparently a counselling charity). This actually meant he was given some leaflets. Thankfully he had enough wish to live to ring us after a cold walk and we provided such support as we are able, as we have for several years.

He had therefore been put through exactly the process of checklist assessment, meaningless non-intervention of ‘weasel words’, legalised neglect, and dangerous and unfair self-guarantee of safety that is described by Beale. This useless approach has therefore, of course, made both him and us feel there is no point in contacting the service again, nor of suggesting anyone else do so.

As doctors and psychiatrists, it has always been our training and teaching that the person/patient was our prime concern, but this is clearly no longer the case. Protection of the system from the rightful needs of patients is the current priority in psychiatric services. The amount of energy and time put into that process now is almost unbelievable and extremely damaging, not only to patients and their families. It is an example of the stigmatisation and dismissal of psychiatric problems, even by staff working in the specialism, that is returning psychiatry to the hopeless laughing stock that it had been before the last war and which we and our teachers and colleagues had done so much to try to turn into a valued and respected medical specialism. Only the sort of fundamental re-humanisation and recovery of professional standards of treatment set out by Beale in her last paragraphs can return psychiatry to self-respect and our patients to proper care.

Beale's paper should be required, albeit uncomfortable reading for all involved in psychiatric care (‘mental health’ are two more weasel words which demean psychiatric illness). Let us hope that Beale's call to arms is heeded for the sake of our patients.
